# Seventeen years after BRCA1: what is the BRCA mutation status of the breast cancer patients in Africa? – a systematic review

**DOI:** 10.1186/2193-1801-1-83

**Published:** 2012-12-28

**Authors:** Lawal AbdulRazzaq Oluwagbemiga, Atoyebi Oluwole, Adesunkanmi AbdulRasheed Kayode

**Affiliations:** 1 Department of Surgery, College of Medicine of the University of Lagos, Lagos, Nigeria; 2 Department of Surgery, College of Health Sciences, Obafemi Awolowo University, Ile Ife, Nigeria

**Keywords:** Breast cancer, Gene mutations, Africa, BRCA1, BRCA2, Systematic review

## Abstract

With the discovery of the BRCA1 gene and other genetic mutations associated with breast cancer, it has been established that hereditary mutations account for up to 5% of patients presenting with breast cancer.

We performed a systematic review of English Language Literature to determine the role of BRCA1 and BRCA2 gene mutations in African breast cancer patients. PUBMED and AJOL database were searched for publications addressing Breast Cancer and BRCA1 and BRCA2 genes. PUBMED was searched using the following words in various combinations; ‘Breast Cancer’, ‘BRCA1’, ‘BRCA2’, ‘BRCA’, ‘Genes’, ‘Cancer Genes’, and ‘Africa’.

16 studies fulfilled the study criteria up till December 2011. The studies were from North Africa (NA) and Sub-Saharan Africa (SSA).

A total of 9 studies were found evaluating 752 (352 repeated Zhang J (2010)) patients from SSA. Three studies (144 patients) evaluated all the coding regions of both BRCA1 and BRCA2 while 2 studies (571 patients) evaluated part(s) of BRCA1 and one (20 Patients) evaluated part(s) of BRCA2, one re-evaluated the whole of the BRCA1 gene in a previous sub-set of patients, while one (16 patients) evaluated parts of both BRCA1 and BRCA2.

In North Africa, 6 studies evaluated 374 patients, with 4 studies (219 patients) evaluating the whole of the BRCA1 and BRCA2 genes while two (155 patients) studies evaluated only parts of both BRCA1 and BRCA2, with one of the studies evaluating the whole of the BRCA1 gene in a subset (24 patients).

Due to this paucity of well powered population based studies evaluating the influence of BRCA genetic mutations in breast cancer patients in Africa, there is a need to perform well powered studies and population screening to determine the impact of germ line mutations in the Breast Cancer patient in Africa before any categorical statements can be made with respect to their BRCA status.

## Introduction

### Epidemiology of breast cancer

Breast cancer is the most common malignancy affecting women worldwide. According to GLOBOCAN there were 1.38 million new cases of breast cancer in the world in 2008, with a corresponding mortality of 458,000. In Africa, over the same time period, there were 68,000 new cases with 37,000 deaths documented, although this probably represents a gross underestimation due to incomplete case ascertainment and reporting Adesunkanmi et al. (2006). In 2010, the World Health Organisation (WHO) estimated that there would be 97,743 new breast cancer cases in Africa, with an estimated mortality of 52,855 IARC (2011).

The reportedly lower incidence of breast cancer in Africa may be explained by lower life expectancy, incomplete record keeping and paucity of epidemiological studies and incomplete case ascertainment. In addition, peculiarities in the presentation of breast cancer in Africa may limit detection Adesunkanmi et al. (2006). These include an apparently younger age and later stage at presentation with more aggressive tumour characteristics Adesunkanmi et al. (2006). The late presentation which may be a consequence of poor health seeking behaviour is also compounded by the more aggressive nature of breast cancer in Africa.

The cause(s) of breast cancer remain largely unknown, though several risk factors have been characterised. The most important factors are the age of the patient and the effect of female sex hormones. Thus early menarche, late menopause and nulliparity all confer an increased risk of breast cancer. Breast feeding is noted to be protective while ingestion of exogenous female sex hormones is a risk factor for breast cancer.

A positive family history of breast cancer is known to increase the risk of breast cancer. However, it was only in 1990, based on the results of linkage analysis performed by Hall et al. (1990) that firm evidence was provided for the existence of at least one hereditary breast cancer gene. Miki et al described the first germ line mutation known to confer increased risk of breast cancer in 1994, labelled BRCA1 Miki et al. (1994). Shortly thereafter, a second germ line mutation associated with increased risk was also identified and named BRCA2 Wooster et al. 1995). Subsequently, several susceptibility genes have been associated with breast cancer. These genes confer ‘high-risk’ and ‘low to moderate risk’ of breast cancer. The high-risk breast cancer susceptibility genes include *BRCA1*, *BRCA2*, *PTEN*, *TP53*, *LKB1/STK11* and *CDH1*, with relative lifetime risks higher than 4 (but generally much higher at young ages). The *CHEK2*, *TGFβ1*, *CASP8* and *ATM* genes belong to the ‘low to moderate-risk’ breast cancer susceptibility genes Oldenburg et al. (2007).

BRCA1 mutation carriers have a 30% risk of developing ovarian cancer during their lifetime Whittemore et al. (1997) and a 50–80% risk of developing breast cancer before the age of 70 years Deng (2006). BRCA1 is mapped to chromosome 17q21 Adesunkanmi et al. (2006); it contains 24 exons and encodes a protein of 220 kDa, composed of 1863 amino acids Chen et al. (1996). The second breast cancer susceptibility gene, BRCA2, is localized on the long arm of chromosome 13. BRCA2 is also a large gene, with 27 exons that encode a protein of 380 kDa, composed of 3418 amino acids Bertwistle et al. (1997).

Risk-associated truncation mutations are found throughout the entire BRCA1 coding sequence Linger & Kruk (2010). The majority of risk-associated mutations are frameshift or nonsense mutations that result in a premature stop codon and truncated protein product (NIH Breast Cancer Information Core Database, http://research.nhgri.nih.gov/bic/). Both BRCA1 and BRCA2 are thought to act as classical tumour suppressor genes and the loss of their cellular functions is thought to occur through bi-allelic inactivation. Carriers of mutations have one germline hit (the inherited mutated copy of BRCA1) and, in the tumour, a second somatic hit usually through the loss of heterozygosity Deng (2006; Collins et al. 1995).

A determination of BRCA genetic mutational status will go a long way towards advice on prophylaxis for breast cancer. The data on BRCA gene mutations in Africans is sparse. The mutational spectrum of BRCA1/2 in African Americans has not been as well characterized as that of Caucasians Ferla et al. (2007).

The knowledge base about the various genetic mutations in the BRCA genes of people of African origin is scattered in diverse small pockets of articles. The aim of this study is to collate all these data and present them in a single review in order to better characterise the genetic mutational spectrum of the indigenous African people.

## Method

Data on breast cancer genetics in Africans was obtained via a literature search. The key words were “*Breast Cancer*” and “*Africa*” with “*Genes*”, “*BRCA Genes*”, “*BRCA1*” and “*BRCA2*” in different combinations. The databases searched were PubMed, African Journals Online (AJOL), the WHO HINARI database, and Ptolemy (University of Toronto library). Only articles published in English were included. References of relevant articles were also reviewed for additional literature. Studies combining and/or comparing Africans with other ethnicgroups were included but only the data of the Africans analysed were included in this review. Studies done outside Africa but which studied an indigenous African people were included.

Specific information retrieved were: type of study, number of exons evaluated, number, type and classification of mutations.

## Result

### BRCA1 and BRCA 2 mutations

#### Sub Saharan African (SSA)

In 1997, Stoppa-Lyonnet Stoppa-Lyonnet et al. (1997) evaluated the whole of the BRCA 1 gene and reported a case of frameshift truncating BRCA1 mutation (926ins10) in a Breast Ovarian Cancer family from Ivory Coast.

Yawitch et al. (2000) evaluated 206 black South African women for 185delAG in exon 2, 4184del4, 943ins10 and 1832deI5 in exon 11, and 5382insC in exon 20, and Met1775Arg in exon 21 mutations in BRCA1. Eleven of these patients had a family history of Breast cancer. None of these mutations were found in any of the patients studied.

Gao Q et al. (2000) screened 70 young African breast cancer patients with early onset breast cancer in Ibadan, Nigeria. Only one of the patients had a family history of breast cancer. All the regions of the BRCA1 and BRCA2 genes were analysed. Eighteen (23%) of the patients had mutations. Three truncating mutations (2 BRCA1 and 1 BRCA2) were detected. Twenty four non truncating mutations (4 BRCA1 and 20 BRCA2) were also detected in this cohort.

Fackenthal et al. (2005) studied 39 Early onset breast cancer (< 40 years) patients in Nigeria. Twenty nine (74%) patients carried a genetic variation in BRCA1 (4 variants), BRCA2 (30 variants) or both genes. However, they found only 1 (3034del4) truncating mutation located on exon 11 of BRCA2. The rest were either unclassified variants or polymorphisms. They went further to test the clinical significance of their finding by examining the BRCA genes of 74 unaffected Nigerians. Five of 13 BRCA2 variations expected to result in deleterious amino acid substitutions were not found in the controls.

A truncating mutation in BRCA1 (Y101X (422 T > G), exon 7) previously identified in Nigerian Breast Cancer patients led Zhang et al. (2009) to screen 365 Nigerian women with Breast cancer and 177 controls for this mutation. This genetic mutation was further discovered in 3 other unrelated patients. All the patients were of the Yoruba ethnic group.

Masri et al. (2002) studied 20 unselected breast cancer patients in Sudan. They analysed Exon 11 of the BRCA2 gene and exon 5–9 of the p53 codon. Four of the patients had a family history of Breast carcinoma, while 1 of the patients – with a family history – had bilateral disease. They found only 1 somatic mutation and 1 polymorphism. They however did not elaborate on the type of mutation and/or the base pairs involved.

Awadelkarim et al. (2007) studied 34 Early onset (< 40 years) and 1 Male Breast Cancer patient in central Sudan. All the coding regions of BRCA1 and BRCA2 were analysed. Majority of the patients (33/35) had mutations. They found 5 truncating mutations (2 in BRCA1, 3 in BRCA2) in 5 patients, one of which, (BRCA2) was in the male patient. They also found 55 unclassified variants (30 in BRCA1, 25 in BRCA2), 5 of which were predicted to affect protein function.

Zhang et al. (2010) evaluated 352 patients from Nigeria previously determined not to have any germline mutations for either BRCA1 or BRCA2 for large genomic rearrangement (LGR). Multiplex ligation-dependent probe amplification (MLPA) was used to screen BRCA1 rearrangements in these patients. They found only 1 patient with a deletion of exon 21.

van der Merwe et al. (2010) screened 16 Black Xhosa patients with breast cancer referred for genetic testing for 6 founder mutations (3 Ashkenazi and 3 Afrikaner). None of these patients carried any of these common founder mutations. They found a mutation on exon 11 of the BRCA2 gene. They then went ahead to screen exon 10 of BRCA1 and exon 10 & 11 of BRCA2. Four of the Xhosa women were found to have a BRCA2 mutation on exon 11. This mutation was also found to be present in 4 out of 105 Colored patients referred for genetic testing.

### North African (NA)

Balci et al. (1999) evaluated BRCA1 and BRCA2 in Turkish breast and ovarian cancer patients with a strong family history of breast cancer in 1999. The whole of the genes were evaluated. In this group of 15 Families of 33 patients, three deleterious mutations (2 BRCA1 and 1 BRCA2) were detected. The BRCA2 mutation was detected in one of the male breast cancer patients.

Ozdag et al. (2000) in 2000 published a study on 50 high risk Turkish Breast cancer patients. Patients were selected based on four criteria; early age at onset, male breast cancer, hereditary breast cancer and familial breast cancer. Exons 2, 5, 11 (10 overlapping fragments), 13, 20 & 24 of BRCA1, and exon 11 (7 overlapping fragments) of BRCA2 were analysed. Four deleterious mutations were found. There were 2 (BRCA2) mutations in the patients with hereditary breast cancer. The other 2 (BRCA1) mutations were found in patients with early onset breast cancer. One of these patients had 2 mutant alleles. There was no truncating mutation discovered in the male breast cancer group. There were 5 BRCA1 sequence variants detected in 23 other patients.

In another Turkish study, Yazici et al. (2000) found 8 BRCA1 and 2 BRCA2 mutations in 11 out of a cohort of 105 patients with breast or ovarian cancer. One (1) of the mutations was found only in an ovarian cancer patient and will not be part of the summary. Half of the patients had a family history while the remaining half had early onset breast cancer (< 50 years). Their analysis was initially limited to exon 11 of both genes and exon 10 of BRCA2. They also performed complete analysis of the BRCA1 gene in a subset of 24 patients and found further mutations in exons 14 and 20, one of which was the Askenazi Jewish mutation 5382insC. This led them to do a heteroduplex analysis of exons 2, 14 and 20 of the BRCA1 gene in all the patients. There was a higher incidence of BRCA mutations (7 BRCA1 and 1 BRCA2) in patients with a family history. Five truncating mutations were identified in exon 11 of both genes. The remaining mutations were in exon 2, 14, 20 and intron 14.

Uhrhammer et al. (2008) performed a study to determine the contribution of BRCA1 mutations to breast cancer in Algeria. The patient population (64) was selected on account of a family history of Breast cancer (13) and early onset Breast cancer (51) (< 38 yrs). They evaluated the whole of the BRCA1 gene. A total of 7 deleterious mutations were found in 11 patients representing 9 families. One of these mutations was found in a patient with early onset breast cancer and another patient with familial breast cancer. Another mutation was found in 2 different families with familial breast cancer.

Troudi et al. (2007) in Tunisia studied 36 patients with family history of breast or ovarian cancer. All of the BRCA1 and BRCA2 genes were analyzed. They found 6 deleterious mutations in 7 patients. Four of these were in BRCA1 (5 patients) and the remainder were in BRCA2 (2 patients). In addition to these mutations, nine distinct unclassified sequence variants (8 in BRCA1 and 1 in BRCA2) and 16 distinct polymorphisms (12 in BRCA1 and 4 in BRCA2) were identified.

Cherbal et al. (2010) studied 86 high risk individuals – male breast cancer, strong family history of breast, ovarian and/or prostate cancers – from 70 families. All the regions of the BRCA1 and BRCA2 genes were evaluated for mutations. Individuals were screened by High-Resolution Melting (HRM) curve analysis followed by direct sequencing. Samples for which no pathogenic mutation was found were analyzed by MLPA. Three pathogenic mutations in *BRCA*1 gene and two within *BRCA2* gene were detected using PCR and HRM. Using MLPA, a novel deletion of *BRCA1* exon 2 and a deletion of *BRCA1* exon 8 were identified in two patients with breast/ovarian cancer and bilateral breast cancer, respectively. The mutations occurred in 8 of 70 families.

Mahfoudh et al. (2011) studied 24 patients in 16 breast/ovarian cancer families. All the coding regions of the BRCA1 gene were analysed. Six families had four deleterious mutations one of which (IVS5 + 2insG) was novel. Another of the mutations (916delTT) was noted in 3 apparently unrelated families.

Tables 1, 2, 3 show a summary of the studies, the portion(s) of the gene(s) evaluated in each study and the mutation(s) detected.

**Table 1:** **Summary of number patients and studies**

Study	Total Pt	Family Hx	Young	Control	BRCA1	BRCA2
		**SUB-SAHARAN AFRICA**				
**Yawitch**	206	11	0	0	00	0
**Gao**	70	1	70	0	2	1
**Fackenthal**	39	1	39	74	1	0
**Zhang B**	365	UNSELECTED		177	1	
**Masri**	20	4				1
**Awadelkarim**	35	(1Male)	34	0	2	3
**Zhang J**	352					
**Stoppa Lyonnet**	NI	1			1	
**Van der Merwe**	16	16				
**TOTAL**	752					
		**NORTH AFRICA**				
**Balci**	33	33 (3 Male)	0	0	0	0
**Ozdag**	50(10 Male)	13	27	0		
**Yazici**	105	53	52	0	7	2
**Urhammer**	64	13	51			
**Troudi**	36	36				
**Cherbal**	86					
Mahfoudh et al. (2011)	24				4	
**TOTAL**	398					
**GRAND TOTAL**	1150

**Table 2:** **Exon(s)/mutations evaluated**

STUDY	BRCA1	BRCA2
**Yawitch**	185delAG in exon 2, 4184del4, 943ins10 and 1832deI5 in exon 11, and 5382insC in exon 20, and Met1775Arg in exon 21	NONE
**Gao**	ALL	ALL
**Fackenthal**	ALL	ALL
**Zhang B**	Y101X (422 T[G), exon 7	NONE
**Masri**	NONE	Exon 11
**Awadelkarim**	ALL	ALL
**Zhang J**	ALL	NONE
**Stoppa Lyonnet**	??	??
**Van der Merwe**	185delAG, 5382insC, 1493delC, 2760 G *>* T, Exon 10	Exon 10 & 11 , 6174delT, 8162delG
	**NORTH AFRICA**	
**Balci**		
Ozdag et al. (2000)	2, 5,11 (10 overlapping fragments), 13, 20, 24	11 (7 overlapping fragments)
**Yazici**	2, 11, 14, 20 (ALL in a subset of patients)	10, 11
Uhrhammer et al. (2008)	ALL	NONE
Troudi et al. (2007)	ALL	ALL
**Cherbal F** (2010)	ALL	ALL
Mahfoudh et al. (2011)	ALL

**Table 3:** **Mutation(s) detected**

STUDY	EXON	MUTATION	EFFECT	CARRIERS	POPULATION
BRCA1					
SUB SAHARAN AFRICA					
Masri	**NOT STUDIED**				
Yawitch	NONE				
Van der Merwe	**NONE**				
Fackenthal et al. (2005)	NONE				
Yazici	2	185 insA			
Cherbal F(2010)	2	Deletion of Exon 2			
Cherbal et al. (2010)	3	c.83_84delTG			
Cherbal et al. (2010)	5	c.181 T *>* G			
Troudi et al. (2007)	5	330 dup A			
Zhang B	7	Y101X (422 T[G)		3	365 (177)
Cherbal et al. (2010)	8	Deletion of Exon 8			
Gao et al. (2000)	11	Q1090X	Stop	1	70
Gao et al. (2000)	11	1742insG	Frameshift	1	70
Stoppa Lyonnet	11	926ins10		1	
Yazici	11	1623delTTAAA			
	11	2139delC			
	11	3819delGTAAA			
Ozdag	11	1201 insA			
	11	K654E			
Troudi et al. (2007)	11	4160 delAG			
	11	2789 del G			
Cherbal et al. (2010)	11	c.798_799delTT			
Stoppa-Lyonnet et al. (1997)	11	926ins10	Frameshift	1	(?160)
Gao et al. (2000)	11	3034/6del4	Frameshift	1	70
Fackenthal et al. (2005)	11	3034/6 delACAA			
Masri	11	Not Indicated			
Yazici	14	4508delC			
	Int 14	IVS-14 + 1delG			
Balci et al. (1999)	20	5382insC	Stop		
Yazici	20	5382insC			
Troudi et al. (2007)	20	5385ins C			
Zhang et al. (2010)	21	Deletion of Exon 21		1	
Balci et al. (1999)	24	5622C – T	Stop		
Awadelkarim KD(2007)	NI	c.3999delT	Stop (codon 1335)		
Awadelkarim et al. (2007)		c.4065_4068delTCAA	Stop (codon 1364)		
Uhrhammer et al. (2008)		c.46_74del29			
		c.83_84delTG			
		c.202 + 1 G > A			
		c.798_799delTT			
		c.1817delC			
		c.2745dupT			
		c.3715delT			
BRCA2					
Yawitch	**NONE**				
Zhang B	**NONE**				
ZHANG J	**NONE**				
Stoppa Lyonnet	**NONE**				
Urhammer	**NONE**				
Troudi et al. (2007)	10	1537 del4			
Cherbal et al. (2010)	10	c.1310_1313delAAGA			
van der Merwe et al. (2010)	11	5999del4 (?Founder)	Frameshift		
Balci et al. (1999)	11	3414delTCAG	Stop		
Ozdag	11	3034 delAAAC			
	11	6880 insG			
Yazici et al. (2000)	11	5295insA			
Yazici et al. (2000)	11	6656delC			
Troudi et al. (2007)	11	5909 ins A			
Cherbal et al. (2010)	11	c.5722 5723delCT			
Awadelkarim et al. (2007)		c.3195_3198delTAAT	Stop (codon 1075)		
Awadelkarim et al. (2007)		c.6406_6407delTT	Stop (codon 2139)		
Awadelkarim et al. (2007)		c.8642_8643insTTTT	Stop (codon 2907)		

NI = Not Indicated, ∞? Same patient as Gao.

## Discussion

Throughout the continent of Africa, over a seventeen year period, 16 studies have evaluated 1150 patients for mutations in the BRCA1 and/or BRCA2 genes in breast cancer patients. Seven hundred and fifty two from SSA and 398 from NA. Four studies evaluated parts of BRCA1 & 2 in 171 patients, 5 studies evaluated the whole of the BRCA1 gene in 465 patients, 6 studies evaluated all the regions of both genes in 299, while 2 studies evaluated parts of BRCA1 in 571 and 1 study evaluated part of BRCA2 in 20 patients.

In this period, the evaluation of genetic mutations in African breast cancer patients has improved from the anecdotal reports of Stoppa-Lyonnet and the evaluation of specific mutations by Yawitch to the discovery of LGRs by Zhang.

In the African breast cancer patients studied, LGRs were found rarely. In some areas, LGRs were also rare in breast and ovarian cancer families de la Hoya et al. (2006; Engert et al. 2008). This contrasts with other areas of the world where Genomic rearrangements account for more than one-third of the brca1 mutations in northern Italian breast/ovarian cancer families Montagna et al. (2003).

## Conclusion

In spite of the lower age at presentation and more aggressive nature of breast cancer in African patients and the association of genetic mutations with breast cancer in young women, there has been a paucity of studies evaluating the genetic basis of breast cancer in this patient population. Evaluation of only 1,150 patients in a disease with a 5 year prevalence of 302,310 at a rate of less than one (1) study per year since the discovery of the BRCA gene is less than adequate. Most of the studies did not evaluate all the coding regions of the genes. Some of them only examined either BRCA1 or BRCA2. Some mutations were found and documented. However, the clinical significance of some of the germ line mutations identified and the clinical application is yet to be confirmed. This is because these studies were grossly underpowered to make any far reaching conclusion about the genetics of the African patient with Breast Cancer. There is therefore a need to perform well powered studies and population screening to determine the impact of germ line mutations in the African Breast Cancer patient.
