# Erratum to: Seventeen years after BRCA1: what is the BRCA mutation status of the breast cancer patients in Africa? – a systematic review

**DOI:** 10.1186/s40064-015-1105-5

**Published:** 2015-07-11

**Authors:** AbdulRazzaq Oluwagbemiga Lawal, Oluwole Atoyebi, AbdulRasheed Kayode Adesunkanmi

**Affiliations:** Department of Surgery, College of Medicine of the University of Lagos, Lagos, Nigeria; Department of Surgery, College of Health Sciences Obafemi Awolowo University, Ile Ife, Nigeria

## Erratum to: SpringerPlus (2012) 1:8 DOI 10.1186/2193-1801-1-83

The article by Lawal et al. (2012), included in the thematic series on Breast Cancer, was published with the authors’ names listed incorrectly.

The names were originally listed as:

‘Lawal AbdulRazzaq Oluwagbemiga, Atoyebi Oluwole and Adesunkanmi AbdulRasheed Kayode’.

The names should instead be listed as follows:

‘AbdulRazzaq Oluwagbemiga Lawal, Oluwole Atoyebi and AbdulRasheed Kayode Adesunkanmi’.

In addition, this article should now be referenced as follows:

Lawal, AO, Atoyebi, O, Adesunkanmi, AK (2012) Seventeen years after BRCA1: what is the BRCA mutation status of the breast cancer patients in Africa? – a systematic review. Springer Plus 1:83.

The publisher apologises for any inconvenience caused.
